# Putative physiological mechanisms underlying tDCS analgesic effects

**DOI:** 10.3389/fnhum.2013.00628

**Published:** 2013-09-26

**Authors:** Helena Knotkova, Michael A. Nitsche, Ricardo A. Cruciani

**Affiliations:** ^1^Department of Pain Medicine and Palliative Care, Institute for Non-Invasive Brain Stimulation, Research Division, Beth Israel Medical CenterNew York, NY, USA; ^2^Department of Neurology, Albert Einstein College of MedicineBronx, NY, USA; ^3^Department of Clinical Neurophysiology, Georg-August-UniversityGoettingen, Germany; ^4^Department of Anesthesiology, Albert Einstein College of MedicineBronx, NY, USA

**Keywords:** transcranial direct current stimulation (tDCS), pain, analgesia, mechanisms, neuromodulation

## Abstract

Transcranial direct current stimulation (tDCS) is a non-invasive neuromodulation technique that induces changes in excitability, and activation of brain neurons and neuronal circuits. It has been observed that beyond regional effects under the electrodes, tDCS also alters activity of remote interconnected cortical and subcortical areas. This makes the tDCS stimulation technique potentially promising for modulation of pain syndromes. Indeed, utilizing specific montages, tDCS resulted in analgesic effects in experimental settings, as well as in post-operative acute pain and chronic pain syndromes. The promising evidence of tDCS-induced analgesic effects raises the challenging and complex question of potential physiologic mechanisms that underlie/mediate the accomplished pain relief. Here we present hypotheses on how the specific montages and targets for stimulation may affect the pain processing network.

Transcranial direct current stimulation (tDCS) is a non-invasive neuromodulation technique (Nitsche and Paulus, 2000) that delivers electrical current of relatively low intensity (1 or 2 miliamperes over an area of about 20 to 35 cm^2^) painlessly through the skull to selected areas of the brain, and induce changes in excitability and activation of brain neurons and neuronal circuits. An important, and perhaps primary, mechanism of tDCS is a subthreshold modulation of neuronal resting membrane potential. Stimulation of several-minute duration results in a polarity-dependent induction of glutamatergic calcium-dependent neuroplasticity, which shares some aspects with long-term potentiation, and depression (Nitsche et al., 2003, 2008). The effects of tDCS on cortical excitability are polarity-dependent. Anodal tDCS enhances, while cathodal tDCS diminishes excitability, within certain parameters of stimulation duration and strength (Nitsche and Paulus, 2000, 2001; Nitsche et al., 2003). Too long, or strong stimulation, however, may have an opposite effect, resulting in diminished excitability after the anodal stimulation and enhanced excitability after the cathodal tDCS (Batsikadze et al., 2013; Monte-Silva et al., 2013). In addition, recent evidence suggests that tDCS interacts with various cerebral neurotransmitter systems, and is mediated by dopamine, acetylcholine, serotonin or GABA (Nitsche et al., 2004a,b,c, 2006, 2009; Kuo et al., 2007; Terney et al., 2008). Moreover, tDCS has been shown to facilitate changes in brain-derived neurotrophic factor (BDNF; Fritsch et al., 2010) that is a distinct marker of neuronal plasticity and notably has been associated with pain processing (Stefani et al., 2012).

The effects and outcome of tDCS depend on the area of the brain that is stimulated (e.g., Nitsche et al., 2007). Beyond regional effects under the electrodes, activity alterations of interconnected remote cortical and subcortical areas have also been described (Polania et al., 2011; DaSilva et al., 2012). This makes the tDCS stimulation technique potentially promising for modulation of pain syndromes, which include pathological alterations of activity, and excitability of a multitude of interconnected areas. Different interwoven cortico-subcortical pain-related networks, so-called Pain Matrix, cover sensory-discriminative, affective, and vegetative aspects of pain processing. The main components of the sensory-discriminative pain processing network are the spinothalamic tract, the lateral thalamus, somatosensory areas, and the posterior insula (Moisset and Bouhassira, 2007). The affective component of pain has been related to anterior insular, and cingulate cortices, as well as prefrontal areas. Vegetative, and neuroendocrine effects of pain perception are closely linked to various subcortical regions, such as amygdala, hypothalamus ventral tegmental area and others (Hsieh et al., 1995; Zaghi et al., 2009). Neuroplastic alterations of connectivity between these areas might contribute to chronification of pain.

Several specific tDCS montages have been probed, which resulted in analgesic effects: (a) excitability-enhancing (anodal) tDCS delivered over the primary motor cortex (e.g., Fregni et al., 2006a,b; Fenton et al., 2008; Kuhnl et al., 2008; Knotkova et al., 2013), typically with the anode positioned over M1 contralateral to the affected side and cathode over the ipsilateral supraorbital region in case of unilateral pain; or the anode over M1 of the dominant hemisphere and the cathode over the supraorbital region contralateral to the anode in case of bilateral pain; (b) excitability-diminishing (cathodal) tDCS over the somatosensory cortex (Antal et al., 2008; Knotkova et al., 2009) [the cathode over S1, the anode over the contralateral supraorbital region, with the same consideration of pain localization as described above]; (c) anodal tDCS over the left dorsolateral prefrontal cortex (DLPFC; Riberto et al., 2011; Valle et al., 2009) [the anode over DLPFC corresponding with the F3 electrode position of the 10–20 international EEG system, the cathode over the contralateral supraorbital region]; (d) combined anodal left DLPFC and cathodal tDCS of contralateral somatosensory cortex [the cathode over the gut representation area of the right S1] (Borckardt et al., 2011).

In the available studies, the assessment of analgesic effects elicited with these montages in subjects with bilateral pain has not systematically compared the pain intensity separately at each side, and thus it is unclear if the effect of the stimulation was unilateral or bilateral. However, as noted by Antal et al. (2010), there is evidence that tDCS of M1 induces widespread changes in cortical activity and can induce changes in activity of the contralateral hemisphere.

Analgesic effects have been explored in experimental settings (experimentally induced pain in healthy subjects), as well as in post-operative acute pain and chronic pain syndromes in clinical settings. The analgesic effects have been shown to be cumulative, and therefore a majority of clinical trials of tDCS encompassed the stimulation on several (usually 5, rarely 10) consecutive days. For example, anodal M1 tDCS over five consecutive days resulted in a significant decrease of pain intensity after spinal cord injury (Fregni et al., 2006a), and similar results were observed in chronic neuropathic pain due to multiple sclerosis (Mori et al., 2010), chronic pelvic pain (Fenton et al., 2009) and pain of various origin (Antal et al., 2010; Knotkova et al., 2013). Stimulation over the prefrontal cortex resulted in significant pain relief after 10 but not 5 sessions (Valle et al., 2009). Meta-analysis of analgesic effects in the existing studies is thoroughly discussed in a Cochrane systematic review by O’Connell et al. (2011). Overall, a significant heterogeneity among studies was noted, and a sub-group evaluation of tDCS applied to the motor cortex suggested superiority of active stimulation over sham (SMD −0.59, 95% CI −1.10 to −0.08).

The evidence of tDCS-induced analgesic effects raises the challenging and complex question of potential physiologic mechanisms that underlie/mediate the accomplished pain relief. Here we develop hypotheses on how the specific montages and targets for stimulation may affect the pain processing network.

## Modulation of the sensory-discriminative pain processing

### Changes in thalamic activity

Thalamic activity is crucial for processing and filtering of nociceptive signals on the pathways ascending to the cortical part of the pain matrix, and the thalamus also receives direct input from descending cortico-thalamic pathways originating in the primary motor cortex. Notably, anodal tDCS over M1 has been shown to increase functional coupling between ipsilateral M1 and thalamus (Polania et al., 2011) and therefore it is likely that the analgesic effects observed after the facilitatory motor cortex stimulation are at least partially attributable to modulation of thalamic activity. Indeed, changes in regional cerebral blood flow following epidural motor cortex stimulation (Peyron et al., 1995; Garcia-Larrea et al., 1999; Garcia-Larrea and Peyron, 2007) indicated that stimulation of the motor cortex may trigger rapid and phasic activation in the lateral thalamus (which receives direct input from the motor area), followed by parallel or secondary activation of medial thalamic regions, and the anterior cingulate gyrus, the insula and the upper brain stem. (Garcia-Larrea et al., 1999). Interestingly, the blood flow change in the lateral thalamus has not significantly correlated with patient’s perceived pain relief and although important as a trigger of further events, the activation of the lateral thalamus is not a sufficient condition for clinical pain-relieving effects (Garcia-Larrea et al., 1999). However, neuronal inactivation in response to motor cortex stimulation was detected in thalamic *sensory* neurons, specifically in ventral posterolateral nuclei and centromedian-parafascicular thalamic complex, and the inactivating effect was particularly observed for neurons responsive to nociceptive peripheral stimulation (Pagano et al., 2012). It can be speculated that the inhibition of the sensory thalamic nuclei and the activation of the lateral (motoric) thalamic area after the motor cortex stimulation may be functionally related, the lateral thalamus receiving the input from the motor cortex and inhibiting the thalamic sensory neurons that are involved in the transmission of nociceptive signals from the periphery.

### Motor-cortex-driven inhibition of the somatosensory cortex

As there is a direct connection between the primary motor cortex and primary somatosensory cortex via cortico-cortical pathways in the human brain, it is possible that stimulation of the motor cortex directly inhibits the activity in the somatosensory cortex. By these means, recent work by Chiou et al. (2012) on animal models demonstrated that motor cortex stimulation blocked the transmission of somatosensory information to the primary somatosensory cortex. In the experiment, epidural motor cortex stimulation, but not stimulation outside of the motor cortex, lead to suppression of the ipsilateral somatosensory evoked potentials. However, these findings have to be interpreted with caution as the stimulation was delivered at suprathreshold level, and therefore the effects cannot be directly extrapolated to the subthreshold tDCS stimulation. Interestingly, a study of the somatosensory cortex in rats (Choi and Callaway, 2011) has shown the existence of inhibitory neurons in the somatosensory cortex that receive direct monosynaptic input not only from distant areas such as thalamus, but also from the ipsilateral motor cortex.

### Direct inhibition of the somatosensory cortex

Cathodal tDCS is thought to have a direct excitability-reducing effect on the S1 area. Since hyperexcitability within S1 in chronic pain syndromes, such as facial neuropathic pain or carpal tunnel syndrome has been clearly documented in recent neuroimaging studies, tDCS-generated reduction of this pathological excitability alteration should be beneficial. Moreover, thickening of neuronal layers in the somatosensory cortex has been observed in chronic migraineurs (DaSilva et al., 2007), which might be a hint for structural neuroplastic alterations of the respective area due to its pain-related hyperactivity of this area. It has been suggested that repeated/long-term down-regulation of nociceptive activity in S1, which could be also induced by tDCS, may result in normalization of this maladaptive change.

## Modulation of the emotional/affective component of pain

### Activation of the prefrontal cortex

Stimulation of the prefrontal cortex has been associated with a modulation of a large neuronal network related to the limbic system, including the cingulate gyrus and parahippocampal areas (Mottaghy et al., 2000; Catafu et al., 2001). The dopaminergic and serotoninergic circuits of the frontal and prefrontal cortex and related subcortical areas mediate attentional control, impulsivity, working memory, decision-making, as well as mood regulation and emotional processing. Notably, activation of the brain structures associated with emotional appraisal of pain in condition of the epidural motor cortex stimulation correlated with subjectively reported pain relief (Peyron et al., 1995; Garcia-Larrea et al., 1999) and it is thought that neuromodulation modifying emotional appraisal of pain and pain experience is directly related to clinical analgesic effects of the neuromodulatory interventions (Garcia-Larrea et al., 1999). Indeed, tDCS stimulation of the prefrontal dorsolateral cortex increased pain thresholds in healthy subjects (Boggio et al., 2008) and relieved chronic pain (Valle et al., 2009).

### Opioid release

 Interestingly, a recent work by DosSantos et al. (2012) has shown that a single session of anodal tDCS over the motor cortex results in reduction of mu opioid receptor binding of an exogenous receptor ligand in the pain matrix, suggesting that the analgesic effect of M1-tDCS may possibly be due to a direct increase of endogenous opioid release (DosSantos et al., 2012). The authors suggest that the decreased binding of the exogenous ligand was possibly due to receptor occupancy by enhanced release of endogenous opioids. The reduction was detected in numerous cortical and subcortical structures of the pain matrix, such as nucleus accumbens, anterior cingulate cortex, insula and thalamus, and was accompanied by an increased threshold for experimentally induced cold pain. Although opioid analgesic effects are known to relate to both the emotional- as well as sensory dimension of pain, no significant changes in clinical pain levels were elicited after a single tDCS session, suggesting that the immediate opiodergic effects of a single tDCS application are subclinical, and repeated application might be necessary to get clinically meaningful results.

## Conclusions and implications for future research

The findings suggest that multiple physiologic mechanisms mediate the analgesic effects of tDCS, involving changes in both the perceptual processing of pain and the emotional component of pain experience. However, the mechanisms and their translation into predicable clinical outcomes are far from being fully understood. Future studies are needed to expand understanding of tDCS-induced analgesic mechanisms and to address the presented hypotheses of tDCS effects on the pain-processing network. Extrapolating from studies of the epidural motor cortex stimulation, changes of the thalamic activation after tDCS may be determined via the regional cerebral blood flow evaluation in the thalamus and related regions after a single- and multiple tDCS stimulation of the motor cortex, including explorations of the association between the thalamic activation changes and pain relief. Future studies addressing the hypothesis of the tDCS-generated analgesic effects due to motor-cortex-driven inhibition of the somatosensory cortex may utilize evaluations of the somatosensory potentials, exploring suppression of the somatosensory evoked potentials after the anodal tDCS stimulation of the ipsilateral motor cortex. Moreover, studies of tDCS combined with functional imaging (fMRI) with regard to the inhibitory (cathodal) stimulation of the somatosensory cortex as well as the anodal tDCS in both the experimentally induced- and spontaneous chronic pain may provide further insight into the tDCS effects on the pain matrix. Beyond the exploration of regional effects, functional imaging data might also be helpful to explore stimulation-dependent alterations of the pain-related cerebral network, via functional connectivity analysis. The latter approach will be also relevant to explore specific effects of different stimulation paradigms on the above-mentioned discernable components of the pain matrix.

Further, future studies are needed to systematically elucidate the impact of the stimulation parameters on the analgesic outcomes, including aspects related to stimulation intensity, strength, repetition rate and timing, as well as electrode positions and stimulation polarity, because a critical aspect of the future impact of tDCS in pain management is the optimization of the stimulation protocols with regard to specific patient populations.

## Conflict of interest statement

The authors declare that the research was conducted in the absence of any commercial or financial relationships that could be construed as a potential conflict of interest.
